# Prevalence and factors associated with complementary feeding practices among children aged 6–23 months in India: a regional analysis

**DOI:** 10.1186/s12889-019-7360-6

**Published:** 2019-08-01

**Authors:** Mansi Vijaybhai Dhami, Felix Akpojene Ogbo, Uchechukwu L. Osuagwu, Kingsley E. Agho

**Affiliations:** 1 Translational Health Research Institute (THRI), School of Medicine, Western Sydney University, Campbelltown Campus, Locked Bag 1797, Penrith, NSW 2571 Australia; 2 General Practice Unit, Prescot Specialist Medical Centre, Welfare Quarters, Makurdi, Benue State Nigeria; 3 School of Medicine Diabetes Obesity and Metabolism Translational Research Unit (DOMTRU), Macarthur Clinical School, Parkside Crescent, Campbelltown, NSW 2560 Australia; 4 School of Science and Health, Western Sydney University, Campbelltown Campus, Locked Bag 1797, Penrith, NSW 2571 Australia

**Keywords:** Complementary feeding, Factors associated, Malnutrition, India, Infant and young child feeding

## Abstract

**Background:**

Inappropriate complementary feeding practices significantly contribute to undernutrition among children under 2 years of age in India. However, there is limited up-to-date evidence on the prevalence and factors associated with complementary feeding practices to guide policy actions at the subnational level in India. We investigated the regional prevalence and factors associated with complementary feeding practices in India.

**Methods:**

This study used a sample of 69,464 maternal responses from the 2015–16 National Family Health Survey in India. The prevalence of complementary feeding indicators was estimated using data for each administrative region, namely: North (*n* = 8469), South (*n* = 12,828), East (*n* = 18,141), West (*n* = 8940), North-East (*n* = 2422) and Central (*n* = 18,664). Factors associated with complementary feeding by region in India were investigated using logistic regression Generalized Linear Latent and Mixed Models (GLLAMM) with a logit link and binomial family that adjusted for clustering and sampling weights.

**Results:**

The study showed a wide variation in the prevalence of introduction of solid, semi-solid or soft foods (complementary foods) among infants aged 6–8 months in regional India; highest in the South (61%) and lowest in the Central and Northern regions (38%). Similarly, minimum dietary diversity (MDD) was highest in the South (33%) and lowest in the Central region (12%). Both minimum meal frequency (MMF) and minimum acceptable diet (MAD) varied substantially across the regions. The factors associated with complementary feeding practices also differed across Indian regions. Significant modifiable factors associated with complementary feeding practices included higher household wealth index for the introduction of complementary foods in the North and Eastern India; higher maternal education for MMF and MDD in the North and Central regions; and frequent antenatal care visits (≥4 visits) for all indicators but for different regions.

**Conclusion:**

Our study indicates that there are wide differences in regional prevalence and factors associated with complementary feeding practices in India. The improvement of complementary feeding practices in India would require national and sub-national efforts that target vulnerable mothers, including those with no education and limited health service contacts.

## Background

Globally, childhood undernutrition is a major public health issue, particularly in developing countries [1]. In 2016, the World Health Organisation (WHO) estimated that 52 million children younger than 5 years were wasted, 17 million were severely wasted, 155 million were stunted and approximately 5% of under 5 deaths could be attributed to malnutrition [2]. Complementary feeding is described as the introduction of safe and nutritionally-balanced solid, semi-solid or soft foods in addition to breast milk for children aged 6–23 months [2]. Appropriate complementary feeding has been linked to optimal childhood nutrition. However, inappropriate complementary feeding practices remain an important aetiology for childhood undernutrition, a major source of disease burden among children under 2 years of age in many developing countries, including India [1, 3].

Appropriate complementary feeding is not only essential for child growth but also provides the foundations required for good health throughout life [2]. Evidence from South Asia suggests that there are wide disparities in complementary feeding practices. For example, India had the lowest proportion of children (15%) who met the minimum dietary diversity (MDD), followed by Nepal (34%) and Bangladesh (42%), while the highest proportion of children who met the MDD was in Sri Lanka (71%) [4]. The study also indicated that India had the lowest percentage of minimum acceptable diet (MAD, 9%) among breastfed children compared with Sri Lankan (68%), Bangladeshi (40%) and Nepalese (32%) children [4]. Although India has taken various steps (e.g., the implementation of the Integrated Child Development Scheme [2] and Anganwadi Services Scheme [5]) to improve the nutritional status of children, evidence suggests that appropriate complementary feeding practices remain low [6, 7]. This indicates the need for additional studies to examine why complementary feeding practices have not improved in the country. 

Previous nationwide studies conducted in India that utilised the 2005–2006 India Demographic and Health Survey (DHS) reported that limited access to health services, low socioeconomic status and no or low maternal education were associated with inappropriate complementary feeding practices [6, 7]. The findings from these studies may not be strictly applicable to the current Indian population due to wide differences in the sample sizes between the 2005–2006 and 2015–2016 India DHS. In the 2015–2016 India DHS, which is the data source for this study, approximately 572,000 households were selected from 1,210.2 million people based on the 2011 census list [8] compared with the 2005–2006 India DHS which selected about 110,000 households from 1,028 million people based on the 2001 census frame [9]. More importantly, the 2015–16 India DHS has been documented to serve as the standard for future nationwide surveys in India [8, 9]. This approach will provide valuable nationally representative data for investigating prevalence and factors associated with various health outcomes (including complementary feeding practices) in India.

To the authors’ knowledge, no past studies have provided a detailed regional analysis of the prevalence of complementary feeding practices, nor has there been an investigation of factors associated with complementary feeding outcomes by region based on the most recent nationally representative data for India (2015–16 India DHS). A detailed regional analysis is needed in India, given the significant disparities in the socioeconomic indicators in the country which has been shown to influence child health and nutritional status [10, 11]**.** The present study aimed to investigate the prevalence and factors associated with complementary feeding practices by region in India.

## Methods

### Data sources

The overall methodology used in this study has been described elsewhere [12]. Briefly, the present study was based on data from the National Family Health Survey 2015–2016 (NFHS-4, also known as the 2015–16 India DHS) which was conducted by the International Institute for Population Sciences, Mumbai, India. A two-stage sampling design was used in both rural and urban areas, where villages and Census Enumeration Blocks were designated as primary sampling units, respectively. Socio-demographic and household characteristics, as well as infant and young child feeding practices data were collected from a sample of women aged between 15 and 49 years. The response rates across the states and territories of India were high, 94.0% in Andhra Pradesh [8] and West Bengal [9], and 99.6% in Bihar [13]. The survey covered 29 states and 7 union territories in India and included approximately 572,000 Indian households. Further information on the sampling procedure used in the NFHS-4 is provided in the respective India DHS state-level reports [8, 9].

### Study population

A total of 69,464 maternal responses for children aged 6–23 months were selected for the survey and were assessed based on the WHO specified complementary feeding indicators. The children were from six administrative regions, namely: North (*n* = 8469), South (*n* = 12,828), East (*n* = 18,141), West (*n* = 8940), Central (*n* = 18,664) and North East (*n* = 2422) regions. The northern region consists of Jammu and Kashmir, Himachal Pradesh, Haryana, Delhi, Chandigarh, Punjab and Rajasthan. Southern region consists of Andaman and Nicobar Islands, Andhra Pradesh, Karnataka, Kerala, Lakshadweep, Puducherry, Tamil Nadu and Telangana. Eastern region consists of Bihar, Jharkhand, Odisha and West Bengal. The western region consists of Gujarat, Maharashtra, Goa, Daman and Diu as well as Dadra and Nagar Haveli. The central region consists of Chhattisgarh, Madhya Pradesh, Uttar Pradesh and Uttarakhand. The north-eastern region consists of Arunachal Pradesh, Assam, Manipur, Meghalaya, Mizoram, Nagaland, Sikkim and Tripura. The distribution of states into regions was performed by the Government of India to facilitate allocation of funds for administrative purposes and improvement of inter-state cooperation [14] and this regional strategy guided our analysis.

### Outcome variables

The assessment of the study outcomes (primary variables) followed the WHO definitions for assessing IYCF indicators in a population [15].

- *Introduction of solid, semi-solid or soft foods (complementary foods):* The proportion of infants 6–8 months of age who received solid, semi-solid or soft foods.
- *Minimum dietary diversity (MDD):* The proportion of children 6–23 months of age who received foods from 4 or more food groups. The 7 food groups used for tabulation of this indicator were: grains, roots and tubers; legumes and nuts; dairy products (milk, yoghurt, cheese); flesh foods (meat, fish, poultry and liver/organ meats), eggs, vitamin-A-rich fruits and vegetables; and other fruits and vegetables.
- *Minimum meal frequency (MMF):* The proportion of breastfed and non-breastfed children 6–23 months of age, who received solid, semi-solid or soft foods (including milk feeds for non-breastfed children) the minimum number of times or more (i.e. 2 times for breastfed infants 6–8 months, 3 times for breastfed children 9–23 months and 4 times for non-breastfed children 6–23 months in the previous day). “Meals” include both meals and snacks (other than trivial amounts), and frequency is based on caregiver report.
- *Minimum acceptable diet (MAD):* The proportion of children 6–23 months of age who received both *minimum dietary diversity* and *minimum meal frequency*. All other indicators were based on a 24-h recall of the infant’s dietary intake by the mother.

### Exposure variables

The explanatory variables were categorised into child, maternal, household, health service and community level characteristics for each region. The characteristics of the child included sex, age, the perceived size of the baby at birth, preceding birth interval and birth order of the child. For the mother, the characteristics included maternal age, education/literacy level, employment status, power over earnings, power over household purchases, as well as the type of caste or tribe and religion.

For the family or household level characteristics, marital status, household wealth index, and access to media sources like newspapers, radio, and television were considered. The household wealth index was derived from a principal component analysis conducted by the IIPS and ICF International and was calculated as a score of ownership of household assets such as transportation device, ownership of durable goods and household facilities. The IIPS and ICF International classified the household wealth index into five categories (quintiles), and each household was assigned to one of these wealth index categories, namely; poorest, poorer, middle, rich and richest [16]. In addition, data on the number of antenatal care (ANC) visits, place of delivery, access to the type of delivery assistance and mode of delivery were considered as health service factors. At the community level, areas of residence (urban or rural) was considered.

### Statistical analysis

The analytical strategy was similar to previously published studies [12, 17, 18]. For this study, preliminary analyses involved the assessment of frequencies and cross-tabulations to calculate the prevalence of complementary feeding practices (i.e., introduction of solid, semi-solid or soft foods, MDD, MMF and MAD) and by the study factors for each geographical region in India. This was followed by an estimation of the prevalence and corresponding confidence intervals of complementary feeding practices by region. Univariable and multivariable logistic regression analyses were conducted to investigate the association between the study variables (child, maternal, household, health service and community factors) and complementary feeding practices using Generalized Linear Latent and Mixed Models (GLLAMM) with a logit link and binomial family that adjusted for clustering and sampling weights.

Specifically, a five-stage modelling methodology was used in the multivariable analyses. First, in modelling of child factors, adjustment for maternal, household, health service and community factors was conducted in the assessment of child factors associated with complementary feeding practices by region in India. We used a similar approach for maternal, household, health service and community factors in the third, fourth and fifth stages, respectively. Adjusted odds ratios (aORs) and their corresponding 95% confidence intervals were calculated and reported as the measure of association between the study factors and complementary feeding practices for each Indian region. All analyses were performed in Stata version 14.0 (Stata Corp, College Station, Texas, USA).

## Results

### Socio-demographic characteristics of the study participants

Across all regions in India, about 80 % of mothers were aged between 25 and 34 years. The majority of mothers in the Central region practised Hindu religion (83.5%) while the religion was the least practised among mothers in the North-Eastern region (46.6%) (Table 1). North-Eastern region had the highest proportion of women (14.8%) who did not receive ANC. More than 70% of mothers across all regions delivered their babies in the health facility, with the highest percentage in the Southern region (96.4%) and the lowest in the North-Eastern region (71.5%) (Table 1).

**Table 1:** Characteristics of the study population by region in India, 2015–16 National Family and Health Survey

Characteristic	North (*n* = 8469)		South (*n* = 12,828)		East (*n* = 18,141)		West (*n* = 8940)		Central (*n* = 18,664)		North Eastern (*n* = 2422)	
	n	%	n	%	n	%	n	%	n	%	n	%
Child Characteristics												
Sex of baby												
Male	4571	54.0	6664	52.0	9443	52.1	4680	52.4	9949	53.3	1254	51.8
Female	3898	46.0	6164	48.1	8698	48.0	4259	47.7	8715	46.7	1168	48.2
Age of child (months)												
6–11	2945	34.8	4427	34.5	6308	34.8	3215	36.0	6761	36.2	787	32.5
12–17	2915	34.4	4262	33.2	6082	33.5	2996	33.5	6120	32.8	876	36.2
18–23	2608	30.8	4139	32.3	5751	31.7	2729	30.5	5782	31.0	760	31.4
Birth order												
First-born	3246	38.3	5547	43.2	6583	36.3	3702	41.4	6091	32.6	978	40.4
2nd-4th	4742	56.0	7176	55.9	10,133	55.9	5020	56.2	10,586	56.7	1263	52.2
5 or more	481	5.7	105	0.8	1425	7.9	217	2.4	1986	10.6	181	7.5
Perceived Size of baby												
Small	871	10.4	1027	8.0	2339	13.1	1043	11.7	2692	14.6	313	13.7
Average	6387	76.4	7976	62.5	11,821	66.0	5711	64.2	13,266	71.9	1493	65.5
Large	1105	13.2	3764	29.5	3742	20.9	2139	24.1	2505	13.6	474	20.8
Preceeding birth interval												
no previous birth	3270	38.6	5604	43.7	6624	36.5	3718	41.6	6130	32.8	981	40.5
< 24 months	1379	16.3	1993	15.5	2833	15.6	1305	14.6	3530	18.9	225	9.3
> 24 months	3820	45.1	5231	40.8	8683	47.9	3916	43.8	9004	48.2	1216	50.2
Maternal Characteristics												
Mother’s age												
15–24 years	231	2.7	569	4.4	1352	7.5	465	5.2	487	2.6	183	7.5
25–34 years	7763	91.7	11,885	92.7	15,607	86.0	8138	91.0	16,874	90.4	2007	82.8
35–49 years	475	5.6	373	2.9	1182	6.5	337	3.8	1303	7.0	233	9.6
Mother’s education												
No education	2406	28.4	1457	11.4	6860	37.8	1183	13.2	6524	35.0	473	19.5
Primary	1217	14.4	1109	8.6	2669	14.7	1072	12.0	2890	15.5	406	16.8
Secondary and above	4846	57.2	10,261	80.0	8612	47.5	6685	74.8	9249	49.6	1543	63.7
Mother’s literacy												
No	5772	68.5	10,873	85.3	10,343	58.0	7268	81.8	11,068	59.6	1818	75.2
Yes	2650	31.5	1874	14.7	7503	42.0	1617	18.2	7505	40.4	599	24.8
Employment												
did not work	1361	88.4	2169	87.0	2673	90.0	1491	82.1	2626	86.3	358	89.4
worked	179	11.6	325	13.0	296	10.0	324	17.9	417	13.7	43	10.6
Mother’s religion												
Hindu	6320	74.6	10,479	81.7	14,093	77.7	7051	78.9	15,587	83.5	1128	46.6
Muslim	1300	15.4	1584	12.4	3508	19.3	1269	14.2	2994	16.0	781	32.2
Christianity and others	849	10.0	764	6.0	540	3.0	620	6.9	83	0.4	514	21.2
Power over earnings												
Husband	7473	88.2	11,302	88.1	16,290	89.8	7862	87.9	16,670	89.3	2145	88.5
Woman	996	11.8	1526	11.9	1851	10.2	1077	12.1	1994	10.7	278	11.5
Power over household purchases												
Husband	7275	85.9	10,908	85	15,817	87.1	7420	83	16,292	87.3	2088	86.2
Woman	1194	14.1	1919	15	2323	12.8	1519	17	2372	12.7	334	13.8
Type of Caste or tribe												
Scheduled caste	2147	25.4	2977	23.2	4117	22.7	1448	16.2	4160	22.3	215	8.9
Scheduled tribe	755	8.9	746	5.8	1904	10.5	1378	15.4	1802	9.7	696	28.7
other backward class	3160	37.3	7187	56.0	7580	41.8	2707	30.3	9466	50.7	450	18.6
other^1^	2407	28.4	1917	15.0	4539	25.0	3406	38.1	3235	17.3	1061	43.8
Family/household characteristics												
Marital status												
Currently married	8421	99.5	12,717	99.2	18,006	99.4	8857	99.2	18,517	99.3	2377	98.3
Formerly married (divorced/separated/widow)	41	0.5	107	0.8	111	0.6	71	0.8	134	0.7	42	1.7
Household Wealth Index												
Poorest	876	10.3	562	4.4	8217	45.3	876	9.8	5784	31.0	593	24.5
Poorer	1296	15.3	1976	15.4	4699	25.9	1612	18.0	4469	23.9	942	38.9
Middle	1711	20.2	3777	29.4	2769	15.3	2192	24.5	3280	17.6	479	19.8
Richer	1930	22.8	3900	30.4	1731	9.5	2324	26.0	2720	14.6	281	11.6
Richest	2656	31.4	2613	20.4	725	4.0	1936	21.7	2411	12.9	127	5.2
Reads newspaper or magazine												
Not at all	5245	61.9	6246	48.7	14,180	78.2	5081	56.8	13,639	73.1	1784	73.6
Yes	3224	38.1	6582	51.3	3961	21.8	3859	43.2	5025	26.9	639	26.4
Listens to radio												
Not at all	7393	87.3	10,955	85.4	15,878	87.5	7775	87.0	16,255	87.1	2077	85.7
Yes	1075	12.7	1873	14.6	2262	12.5	1165	13.0	2409	12.9	346	14.3
Watches television												
Not at all	1669	19.7	841	6.6	8786	48.4	1536	17.2	7134	38.2	893	36.9
Yes	6799	80.3	11,986	93.4	9354	51.6	7403	82.8	11,530	61.8	1530	63.2
Health Service characteristics												
Antenatal clinic visits												
None	961	11.4	806	6.3	4719	26.0	862	9.6	3789	20.3	357	14.8
1–3	3121	36.9	2038	15.9	6133	33.8	1595	17.8	8848	47.4	870	35.9
4+	4386	51.8	9983	77.8	7289	40.2	6483	72.5	6026	32.3	1195	49.4
Place of delivery												
Home	1002	11.8	466	3.6	4734	26.1	715	8.0	4615	24.7	691	28.5
Health facility	1767	88.2	12,362	96.4	13,407	73.9	8224	92.0	14,049	75.3	1731	71.5
Type of delivery assistance												
Health professional	6899	81.7	11,477	89.6	11,130	61.9	7031	79.2	10,710	57.7	1694	71.2
Traditional birth attendants.	598	7.1	274	2.1	2873	16.0	320	3.6	2282	12.3	267	11.2
Other untrained	946	11.2	1060	8.3	3984	22.2	1525	17.2	5557	30.0	420	17.7
Mode of delivery												
Vaginal	6976	82.4	7795	60.8	15,472	85.3	7006	78.4	16,708	89.5	2046	84.4
Caesarean	1492	17.6	5033	39.2	2669	14.7	1934	21.6	1956	10.5	377	15.6
Community level factor												
Residence												
Urban	2881	34.0	5265	41.0	3046	16.8	3869	43.3	4191	22.5	347	14.3
Rural	5588	66.0	7563	59.0	15,095	83.2	5071	56.7	14,472	77.5	2076	85.7

*n* = weighted counts

other^1^ - Includes Jews, Parsis/Zoroastrians, those following “other” religions, and those with no religion

### Regional prevalence of complementary feeding indicators

**Fig. 1:**
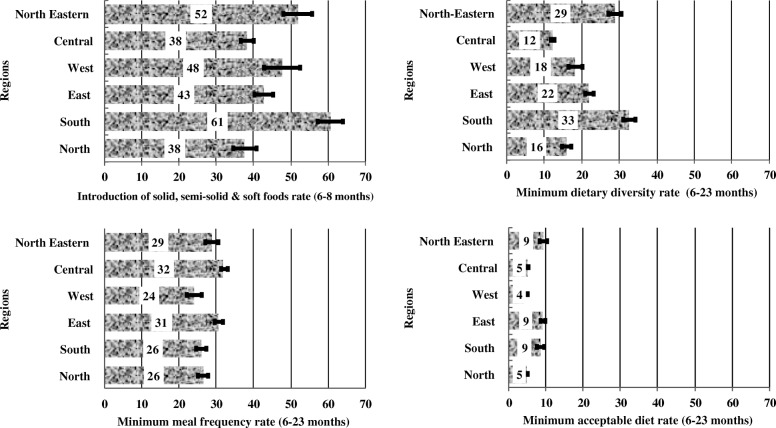
Regional prevalence of complementary feeding practices in India, 2015–16 National Family and Health Survey

Southern region had the highest prevalence of complementary feeding among infants aged 6–8 months compared with other regions. Both Southern (33%) and North-Eastern (29%) regions had better MDD prevalence among children aged 6–23 months compared with other regions, whereas the Central region had the lowest prevalence (12%) (Fig. 1). Western region had the lowest MMF prevalence compared with North-Eastern, Central and Eastern regions (24 vs 29, 32, 31%, respectively). Similarly, Western (4%), Central (5%) and Northern (5%) regions had the lowest prevalence of MAD compared with North-Eastern, Eastern and Southern regions (9% each) (Fig. 1)

### Factors associated with the introduction of solid, semi-solid or soft foods by region

Western urban women were more likely to introduce complementary foods compared with Western rural women (Table 2) In the Central region, second to fourth born children and those in the birth order of five or more were more likely to be introduced to complementary foods compared with first-born children. In the Eastern and Central regions, mothers who had frequent ANC visits were more likely to introduce complementary foods to their infants compared with those who made no ANC visits, while mothers from the North-Eastern region who listened to the radio and watched the television were more likely to introduce their infants to complementary foods compared with those who did not listen to the radio and/or watch the television (Table 2)

**Table 2:** Factors associated with introduction of solid, semi-solid or soft foods in India, 2015–16 National Family and Health Survey

Characteristics	North	South	East	West	Central	North Eastern
	aOR[95% CI]	aOR [95% CI]	aOR [95% CI]	aOR [95% CI]	aOR [95% CI]	aOR [95% CI]
Residence						
Rural				1.00		
Urban				1.51 (1.10, 2.07)^*^		
Type of Caste or tribe						
Scheduled Caste	1.00					1.00
Scheduled tribe	0.62 (0.43, 0.88)^*^					1.69 (1.09, 2.61) ^*^
Other backward class	0.90 (0.73, 1.12)					1.18 (0.71, 1.98)
Others	1.02 (0.82, 1.28)					1.42 (0.88, 2.29)
Household Wealth Index						
Poorest	1.00		1.00			
Poorer	1.42 (0.99, 2.04)		1.26 (1.03, 1.53)^*^			
Middle	1.38 (0.96, 1.96)		1.24 (0.96, 1.61)			
Richer	1.50 (1.06, 3.13)^*^		1.15 (0.85, 1.55)			
Richest	1.72 (1.23, 1.92)^*^		1.23 (0.78, 1.92)			
Mother’s education						
No education					1.00	
Primary					1.09 (0.91, 1.32)	
Secondary and above					1.18 (1.02,1.37)^*^	
Mother’s age						
15–24 years				1.00	1.00	
25–34 years				0.53 (0.27, 1.02)	0.41 (0.31, 0.54)^*^	
35–49 years				0.22 (0.08, 0.64)^*^	0.33 (0.22, 0.51)^*^	
Power over earning						
Husband	1.00	1.00	1.00	1.00	1.00	1.00
woman	0.94 (0.72, 1.24)	1.02 (0.78, 1.34)	1.04 (0.80, 1.36)	1.29 (0.84, 1.98)	0.84 (0.69, 1.01)	1.23 (0.83, 1.83)
Power over household purchases						
Husband	1.00	1.00	1.00	1.00	1.00	1.00
woman	1.01 (0.79, 1.29)	1.03 (0.80, 1.32)	1.15 (0.90, 1.48)	1.43 (0.96, 2.13)	0.86 (0.72, 1.03)	1.24 (0.88, 1.77)
Birth order						
First-born					1.00	
2nd-4th					1.28 (1.11, 1.6)^*^	
5 or more					1.47 (1.14, 1.89)^*^	
Type of delivery assistance						
Health professional		1.00				
Traditional birth attendant		0.30 (0.12, 0.78)^*^				
Other untrained		0.67 (0.45, 1.00)				
Antenatal clinic visits						
None			1.00		1.00	
1–3			1.01 (0.82, 1.25)		1.38 (1.15, 1.64) ^*^	
4+			1.38 (1.12, 1.72)^*^		1.80 (1.49, 2.17) ^*^	
Listens to radio						
Not at all						1.00
Yes						1.48 (1.15, 1.92) ^*^
Watches television						
Not at all						1.00
Yes						1.30 (1.01, 1.69) ^*^

Statistically significant (95% confidence intervals and *P* < 0.05^*^) study variables from multivariable models are shown. In the model of child factors, adjustments were made for maternal, household, health service and community factors. A similar approach was used for maternal, household, health service and community factors that adjusted for respective factors in multivariable models

### Factors associated with MMF by region

Children of mothers from the richest household category in the Southern and Eastern regions were more likely to meet MMF compared with those in the lower household wealth category (Table 3). Higher maternal education (secondary and higher education) increased the odds of children meeting MMF in the Northern, Southern and Central regions compared with those with no education. Woman autonomy over finances and household decision making were associated with MMF in the Central region. Second to fourth born children were more likely to meet the MMF compared with first-born children in the Northern, Eastern, Central and North Eastern regions. Infants of mothers who had TBA- and health professional-assisted births were more likely to meet the MMF compared with those who delivered at home with untrained personnel in the Eastern region. Mothers in the Northern, Southern, Eastern and Central regions who had four or more ANC visits were more likely to have children who met the MMF compared with those who made no ANC visits (Table 3).

**Table 3:** Factors associated with minimum meal frequency in India, 2015–16 National Family and Health Survey

Characteristics	North	South	East	West	Central	North Eastern
	aOR [95% CI]	aOR [95% CI]	aOR [95% CI]	aOR [95% CI]	aOR [95% CI]	aOR [95% CI]
Type of Caste or tribe						
Scheduled Caste	1.00	1.00	1.00		1.00	1.00
Scheduled tribe	1.11 (0.90, 1.37)	0.86 (0.61, 1.21)	1.08 (0.91, 1.28)		1.36 (1.19, 1.55) ^*^	1.57 (1.20, 2.05) ^*^
Other backward class	1.10 (0.96, 1.27)	0.87 (0.73, 1.04)	0.85 (0.75, 0.96) ^*^		1.02 (0.92, 1.12)	1.15 (0.85, 1.55)
Others	1.19 (1.02, 1.38) ^*^	0.77 (0.61, 0.98)^*^	0.79 (0.66, 0.94) ^*^		0.94 (0.84, 1.05)	1.05 (0.79, 1.39)
Household Wealth Index						
Poorest		1.00	1.00			
Poorer		1.24 (0.86, 1.80)	1.02 (0.90, 1.15)			
Middle		1.22 (0.85, 1.75)	1.00 (0.85, 1.17)			
Richer		1.13 (0.78, 1.65)	0.98 (0.80, 1.20)			
Richest		1.64 (1.10, 2.45) ^*^	1.41 (1.01, 1.96) ^*^			
Mother’s religion						
Hindu			1.00			
Muslim			1.03 (0.88, 1.21)			
Christianity and others			1.35 (1.04, 1.75) ^*^			
Marital status						
Currently married			1.00		1.00	
Formerly married (divorced / separated / widow)			1.73 (1.10, 2.72) ^*^		1.61 (1.10, 1.27) ^*^	
Mother’s education						
No education	1.00	1.00				
Primary	1.16 (0.97, 1.37)	0.97 (0.70, 1.37)			1.08 (0.97, 1.20)	
Secondary and above	1.24 (1.08, 1.42) ^*^	1.31 (1.02, 1.67) ^*^			1.15 (1.04, 1.27) ^*^	
Power over earning						
Husband	1.00	1.00	1.00	1.00	1.00	1.00
woman	1.10 (0.93, 1.29)	1.04 (0.85, 1.27)	1.00 (0.85, 1.17)	1.20 (0.88, 1.63)	1.15 (1.03, 1.29) ^*^	1.09 (0.89, 1.33)
Power over Household purchases						
Husband	1.00	1.00	1.00	1.00	1.00	1.00
woman	1.11 (0.96, 1.29)	1.07 (0.89, 1.29)	1.09 (0.94, 1.26)	1.27 (0.97, 1.66)	1.13 (1.01, 1.25) ^*^	1.16 (0.96, 1.40)
Birth order						
First-born	1.00		1.00		1.00	1.00
2nd-4th	1.27 (1.14, 1.42) ^*^		1.23 (1.11, 1.36) ^*^		1.23 (1.14, 1.33) ^*^	1.26 (1.09, 1.46) ^*^
5 or more	1.45 (1.14, 1.86) ^*^		1.14 (0.96, 1.37)		1.32 (1.16, 1.51) ^*^	1.44 (1.13, 1.84) ^*^
Type of delivery assistance						
Health professional		1.00	1.00		1.00	
Traditional birth attendant		0.36 (0.16, 0.80) ^*^	1.44 (1.24, 1.67) ^*^		0.99 (0.89, 1.12)	
Other untrained personnel		0.80 (0.62, 1.04)	1.45 (1.27, 1.65) ^*^		1.24 (1.14, 1.35) ^*^	
Antenatal clinic visits						
None	1.00	1.00	1.00		1.00	
1–3	1.27 (1.02, 1.59) ^*^	1.09 (0.76, 1.59)	1.09 (0.96, 1.23)		1.22 (1.10, 1.35) ^*^	
4+	1.65 (1.31, 2.06) ^*^	1.74 (1.27, 2.38) ^*^	1.49 (1.30, 1.72) ^*^		1.66 (1.48, 1.86) ^*^	
Place of delivery						
Home	1.00		1.00			
Health facility	0.77 (0.64, 0.93) ^*^		1.27 (1.11, 1.44) ^*^			

Statistically significant (95% confidence intervals and *P* < 0.05^*^) study variables from multivariable models are shown. In the model of child factors, adjustments were made for maternal, household, health service and community factors. A similar approach was used for maternal, household, health service and community factors that adjusted for respective factors in multivariable models

### Factors associated with MDD by region

In the Northern, Western, Central and North Eastern regions, children whose mothers belonged to the richest household wealth category had higher odds of meeting MDD compared with those from other regions and quintiles (Table 4). Infants of mothers from the Northern and Central regions who had secondary and higher education were more likely to meet the MDD compared with infants of mothers with no education. Mothers from the Northern and Central regions who were aged 35–49 years had higher odds of having children who met MDD compared with their counterparts. Mothers from the Southern region who had power over earnings and household purchases were more likely to have children who met MDD compared with their counterparts in other regions. The study found that second to fourth born children in the North, South, East, West and North Eastern regions were more likely to have diverse diet compared with first-born children in these regions. Mothers who had four or more ANC visits in the Eastern region were more likely to have children who met the MDD compared with those who had no ANC visits (Table 4).

**Table 4:** Factors associated with minimum dietary diversity in India, 2015–16 National Family and Health Survey

Characteristics	North	South	East	West	Central	North Eastern
	aOR [95% CI]	aOR [95% CI]	aOR [95% CI]	aOR [95% CI]	aOR [95% CI]	aOR [95% CI]
Type of Caste or tribe						
Scheduled Caste	1.00		1.00		1.00	
Scheduled tribe	1.12(0.82,1.51)		1.02(0.83,1.26)		1.50(1.24,1.81) ^*^	
Other backward class	0.71(0.59,0.85) ^*^		0.85(0.73,0.99) ^*^		1.02(0.89,1.17)	
Others	1.09(0.90,1.31)		1.24(1.01,1.50)		1.02(0.86,1.21)	
Household Wealth Index						
Poorest	1.00			1.00	1.00	1.00
Poorer	1.46(1.07,2.00) ^*^			1.06(0.71,1.60)	1.17(1.01,1.35) ^*^	1.05(0.84,1.32)
Middle	2.19(1.61,2.99) ^*^			0.93(0.65,1.40)	1.35(1.13,1.61) ^*^	1.06(0.84,1.35)
Richer	2.34(1.70,3.23) ^*^			1.68(1.10,2.54) ^*^	1.45(1.21,1.74) ^*^	1.42(1.06,1.91) ^*^
Richest	2.54(1.82,3.55) ^*^			1.62(1.06,2.52) ^*^	1.66(1.36,2.02) ^*^	1.52(1.07,2.18) ^*^
Mother’s religion						
Hindu	1.00	1.00	1.00			1.00
Muslim	2.36(1.96,2.83) ^*^	0.70(0.56,0.88) ^*^	1.32(1.10,1.58) ^*^			0.85(0.68,1.05)
Christianity and others	0.88(0.70,1.10)	0.89(0.65,1.22)	0.97(0.70,1.34)			1.58(1.35,1.85) ^*^
Mother’s education						
No education	1.00				1.00	
Primary	0.89(0.69,1.14)				1.16(0.94,1.43)	
Secondary and above	1.42(1.15,1.75) ^*^				1.46(1.13,1.90) ^*^	
Mother’s literacy						
No		1.00		1.00		
Yes		0.46(0.37,0.57)^*^		1.28(1.01,1.62) ^*^		
Mother’s age						
15–24 years	1.00				1.00	
25–34 years	2.25(1.41,3.58) ^*^				1.41(1.04,1.91) ^*^	
35–49 years	2.61(1.15,1.75) ^*^				1.63(1.14,2.33) ^*^	
Power over earning						
Husband	1.00	1.00	1.00	1.00	1.00	1.00
Woman	1.09(0.89,1.33)	1.29(1.07,1.56) ^*^	1.13(0.93,1.37)	1.14(0.77,1.68)	1.01(0.86,1.19)	0.89(0.72,1.11)
Power over household purchases						
Husband	1.00	1.00	1.00	1.00	1.00	1.00
Woman	1.13(0.94,1.36)	1.26(1.05,1.51) ^*^	1.08(0.91,1.29)	0.91(0.64,1.29)	0.98(0.85,1.14)	0.86(0.70,1.05)
Birth order						
First-born	1.00	1.00	1.00	1.00		1.00
2nd-4th	1.15(1.01,1.32) ^*^	1.15(1.01,1.31) ^*^	1.26(1.11,1.43) ^*^	1.36(1.08,1.72) ^*^		1.41(1.21,1.64) ^*^
5 or more	0.76(0.52,1.10)	0.90(0.47,1.71)	1.08(0.86,1.36)	1.10(0.57,2.14)		1.05(0.78,1.40)
Perceived Size of baby						
Small	1.00	1.00		1.00	1.00	1.00
Average	0.77(0.62,0.96) ^*^	0.76(0.61,0.96) ^*^		0.65(0.46,0.92) ^*^	1.22(1.05,1.41) ^*^	0.75(0.59,0.94) ^*^
Large	0.83(0.63,1.10)	0.83(0.65,1.07)		0.82(0.57,1.18)	1.40(1.16,1.69) ^*^	0.98(0.75,1.28)
Type of delivery assistance						
Health professional	1.00	1.00		1.00	1.00	
Traditional birth attendants	1.15(0.86,1.54)	0.26(0.12,0.53) ^*^		1.15(0.98,1.35) ^*^		
Other untrained personnel	1.42(1.15,1.75) ^*^	1.02(0.81,1.29)			1.16(1.03,1.31) ^*^	
Antenatal clinic visits						
None	1.00	1.00	1.00		1.00	
1–3	0.72(0.55,0.95) ^*^	0.59(0.42,0.82) ^*^	0.85(0.74,0.98) ^*^		0.81(0.70,0.94) ^*^	
4+	0.90(0.69,1.19)	0.75(0.56,0.99) ^*^	1.26(1.08,1.48) ^*^		1.01(0.86,1.18)	
Listens to radio						
Not at all	1.00				1.00	
Yes	1.27(1.05,1.52) ^*^				1.19(1.03,1.37) ^*^	
Watches television						
Not at all			1.00			
Yes			1.31(1.15,1.49) ^*^			
Reads newspaper or magazine						
Not at all				1.00	1.00	1.00
Yes				1.50(1.15,1.95) ^*^	1.15(1.00,1.32) ^*^	1.27(1.08,1.50) ^*^

Statistically significant (95% confidence intervals and *P* < 0.05^*^) study variables from multivariable models are shown. In the model of child factors, adjustments were made for maternal, household, health service and community factors. A similar approach was used for maternal, household, health service and community factors that adjusted for respective factors in multivariable models

### Factors associated with MAD by region

Mothers from wealthier households in the Northern and Southern regions were more likely to have children who met MAD compared with mothers from poorer households (Table 5). Similarly, older mothers (≥ 25 years) in the Northern region were more likely to have children who met MAD compared with younger mothers (< 25 years). Second to fourth born children in the Northern, Eastern, Central and North-Eastern regions were also more likely to meet MAD compared with first-born children in these regions. Children in the Southern region who were delivered in health facilities were more likely to meet MAD compared with those delivered at home. Mothers who had four or more ANC visits in Eastern and Central regions were more likely to have children who met MAD compared with those who had no ANC visit (Table 5).

**Table 5:** Factors associated with minimum acceptable diet in India, 2015–16 National Family and Health Survey

Characteristics	North	South	East	West	Central	North Eastern
	aOR [95% CI]	aOR [95% CI]	aOR [95% CI]	aOR [95% CI]	aOR [95% CI]	aOR [95% CI]
Type of Caste or tribe						
Scheduled Caste	1.00	1.00	1.00		1.00	
Scheduled tribe	1.52(0.98,2.34)	0.78(0.47,1.30)	0.88(0.66,1.18)		1.43(1.11,1.85) ^*^	
Other backward class	0.96(0.70,1.31)	0.67(0.52,0.86) ^*^	0.76(0.61,0.93) ^*^		1.09(0.90,1.32)	
Others	1.48(1.10,1.98)^*^	0.71(0.46,1.11)	1.11(0.85,1.45)		1.10(0.87,1.38)	
Household Wealth Index						
Poorest	1.00	1.00				
Poorer	1.27(0.75,2.15)	1.29(0.71,2.32)				
Middle	1.52(0.91,2.52)	1.75(0.99,3.09)				
Richer	1.68(0.98,2.85)	1.50(0.84,2.68)				
Richest	1.81(1.05,3.10) ^*^	2.60(1.40,4.81) ^*^				
Mother’s religion						
Hindu	1.00		1.00			1.00
Muslim	2.00(1.58,2.52) ^*^		1.18(0.92,1.52)			0.83(0.60,1.14)
Christianity and others	0.94(0.62,1.39)		1.59(1.08,2.35) ^*^			1.95(1.58,2.42) ^*^
Marital status						
Currently married				1.00		
Formerly married (divorced / separated /widowed)				0.05(0.01,0.38) ^*^		
Mother’s education						
No education					1.00	
Primary					1.09(0.86,1.37)	
Secondary and above					1.29(1.08,1.56) ^*^	
Mother’s literacy						
No	1.00					
Yes	0.69(0.53,0.90) ^*^					
Mother’s age						
15–24 years	1.00					
25–34 years	2.24(1.07,4.66) ^*^					
35–49 years	2.73(1.19,6.26) ^*^					
Power over earning						
Husband	1.00	1.00	1.00	1.00	1.00	1.00
woman	0.89(0.68,1.17)	1.06(0.79,1.42)	1.09(0.82,1.44)	1.22(0.69,2.13)	1.16(0.92,1.45)	1.09(0.82,1.46)
Power over household purchases						
Husband	1.00	1.00	1.00	1.00	1.00	1.00
woman	0.98(0.76,1.26)	1.17(0.89,1.54)	1.06(0.82,1.36)	1.04(0.62,1.73)	1.05(0.86,1.30)	1.11(0.84,1.47)
Sex of baby						
Male					1	
Female					0.86(0.74,0.99) ^*^	
Birth order						
First-born	1.00		1.00		1.00	1.00
2nd-4th	1.27(1.03,1.56) ^*^		1.41(1.17,1.69) ^*^		1.24(1.05,1.46) ^*^	1.41(1.14,1.74) ^*^
5 or more	1.09(0.65,1.82)		1.20(0.87,1.66)		1.21(0.90,1.63)	1.19(0.85,1.65)
Perceived Size of baby						
Small				1.00	1.00	
Average				0.50(0.26,0.96) ^*^	1.26(1.00,1.60) ^*^	
Large				0.54(0.26,1.10)	1.29(0.96,1.72)	
Type of delivery assistance						
Health professional			1.00		1.00	
Traditional birth attendants			1.26(1.00,1.58) ^*^		1.07(0.84,1.37)	
Other untrained			1.24(0.99,1.54)		1.30(1.09,1.56) ^*^	
Place of delivery						
Home		1.00				
Health facility		2.77(1.18,6.50) ^*^				
Antenatal clinic visits						
None			1.00		1.00	
1–3			0.79(0.64,0.99) ^*^		1.04(0.84,1.29)	
4+			1.63(1.31,2.04) ^*^		1.57(1.25,1.98) ^*^	
Listens to radio						
Not at all	1.00			1.00	1.00	
Yes	1.39(1.07,1.79) ^*^			0.45(0.25,0.82) ^*^	1.45(1.19,1.77) ^*^	
Watches television						
Not at all			1.00			
Yes			1.58(1.31,1.90) ^*^			
Reads newspaper or magazine						
Not at all						1.00
Yes						1.40(1.14,1.71) ^*^

Statistically significant (95% confidence intervals and *P* < 0.05^*^) study variables from multivariable models are shown. In the model of child factors, adjustments were made for maternal, household, health service and community factors. A similar approach was used for maternal, household, health service and community factors that adjusted for respective factors in multivariable models

## Discussion

The present study found significant regional differences in the prevalence of complementary feeding practices among infants and young children aged 6–23 months in India, with the highest prevalence observed in the Southern and North-Eastern regions. Similarly, the analyses showed wide regional variations in the factors associated with complementary feeding practices in India, possibly reflecting differences in the socio-economic and cultural characteristics previously reported in Indian communities [10, 11, 19, 20]. Birth order was one of the most consistent factors associated with MDD, MMF and MAD in most regions. Key modifiable factors (higher household wealth status, higher maternal education, skilled-assisted deliveries and frequent ANC visits) that were associated with complementary feeding practices also varied across the regions in India.

Higher maternal education was associated with the introduction of complementary foods among mothers from the Southern, Central and Northern regions in India. This finding was consistent with previous reports from Ghana [21], Ethiopia [22–24], and five European countries [25]. However, studies from Nepal [26] and Lebanon [27] suggested that there was no significant association between higher maternal education and complementary feeding practices. The relationship between higher maternal education and appropriate complementary practices could be due to the promotion of optimal maternal and child nutrition, and subsequent uptake of health information and health-seeking behaviour of educated mothers [28]. The present study also indicated that higher household wealth status was strongly associated with MDD, MMF and MAD, with marked variations across the regions in India. This finding possibly demonstrates the impact of household socioeconomic variability on childhood nutrition in many Indian communities [29]. Evidence from Pakistan [30] has suggested that there was a strong relationship between maternal education and socioeconomic status with malnutrition among children younger than 5 years, where educated mothers demonstrated better knowledge of child nutrition and; they were more likely to translate that information into practice [28].

In this study, frequent ANC (≥4) visits were associated with the introduction of complementary foods to infants of mothers from the Eastern and Central regions. Mothers who had at least four ANC visits were more likely to introduce complementary foods and have children who were more likely to meet the MDD compared with mothers who had no ANC visits. These findings may be attributed to a number of factors relating to non-use of ANC, including low maternal education, the cost associated with attending ANC service and a lack of awareness of the importance of ANC among mothers [31–34]. Similarly, a study conducted in India indicated that lower ANC visits were related to delayed introduction of complementary foods [6]. Studies from Nepal [35] and Ethiopia [24] found a similar influence of ANC visits on the introduction of complementary feeding practices. In Pakistan, however, fewer ANC visits increased the odds of meeting MMF in children [36]. The identification of relevant health service factors for specific health outcomes has implications for improving health outcomes among vulnerable populations. Therefore, effective nutrition education and counselling (often provided during ANC visits) are needed in many Indian communities and should focus on at-risk regions and populations to maximise impacts of nutritional interventions (such as improving mothers’ infant feeding knowledge and practices) [37, 38]. In Bangladesh, for example, intensive counselling that was combined with a nationwide mass media campaign substantially improved the proportion of children who met MAD from 16 to 50%, an improvement of 68% [39]. Moreover, the involvement of various movie stars to promote appropriate complementary feeding practices through audio-visual media in Indian communities has been flagged as a strategy that needs to be explored to improve complementary feeding practices [40].

The present study showed that approximately 16% of mothers received assistance from a health professional during delivery. The small proportion of mothers who received skilled-assisted delivery compared to previous regional reports (50%) [16, 41], is possibly a reflection of the high prevalence of home deliveries in India [16]. This study also showed that skilled-assisted delivery increased the odds of introducing complementary foods to infants compared with mothers who had no skilled-assisted delivery in the Southern region of India, likely reflecting one of the importance of skilled-assisted birthing. In contrast, non-skilled assisted delivery increased the odds of meeting the MDD and MAD among infants of mothers from the East and Central regions in India. This indicates that women delivering in health facilities may not have been appropriately counselled about appropriate IYCF options or it could be a reflection of the availability and accessibility of local food types for infants. Mothers who had skilled-assisted delivery should benefit fully from the counselling provided by health professionals that involved IYCF practices [42]. In addition, skilled-assisted delivery offers the mother a unique opportunity to access appropriate child feeding information which can improve the mother’s capacity to challenge unfavourable information and infant feeding attitudes in the community [43]. Improving maternal health capacity and resources (such as increased skilled-assisted delivery for mothers) across Indian regions would not only have a significant impact on maternal health but also on complementary feeding practices.

Another finding of this study was that children in higher birth order (second and above) were more likely to be introduced to complementary feeding compared with those in the first-birth order, irrespective of the Indian region. A previous study conducted among Ethiopian women found significant associations between higher birth order and MDD [44]. Similarly, a community-based study found that higher birth order was associated with MAD [27], suggesting that an increased level of awareness of appropriate complementary feeding practices can be gained through experience from previous childbirth. Mothers became more experienced after nurturing their first babies, and this can possibly be translated to improve complementary feeding practices [27]. This implies that efforts aimed at improving complementary feeding practices in India should also target nulliparous mothers to maximise results of IYCF interventions.

The study indicated that higher maternal age (≥25 years) was associated with complementary feeding practices, with variations across the regions in India. Specifically, while older mothers were less likely to introduce complementary foods to their infants in the West and Central regions, Northern and Central, older mothers were more likely to have infants who met the MDD and MAD compared to younger mothers (< 25 years) in those regions. Such significant effects of maternal age on complementary feeding suggests that the mother’s experience may play a significant role in appropriate IYCF practices. Our findings are consistent with evidence from Nepal which indicated that children of older mothers (≥35 years) were more likely to meet MDD and MAD compared with children of younger mothers (< 20 years) [35]**.** Our study findings imply that IYCF policy and interventions should also focus on younger mothers who are likely to be primiparous women with little experience in IYCF practices.

The present study also indicated that women autonomy over household earnings and power over household purchases were associated with complementary feeding practices, with demonstrable regional variations across India. Specifically, our study demonstrated that children of mothers who had power over household earnings and decision making were more likely to meet MMF compared to their counterparts from Central India. Similar findings were observed in the Southern region for MDD. These results may be attributable to high maternal education, the freedom to exchange information and interact with other people outside of the family at social gatherings or markets and exposure to media [45, 46]. Also, higher power over the financial resources and mass media exposure may indicate a higher likelihood of better resources allocated for child nutrition [26, 45, 47, 48]. Our findings were consistent with various regional studies in India which indicated that maternal autonomy was associated with childhood nutrition [49–51].

Various methodological limitations need to be considered when interpreting the study results. First, the information on the survey was based on interviews and subjective responses from mothers, and the results may be affected by recall bias. However, the analyses were restricted to the most recent child, alive and living with the respondent to reduce the potential effect of recall bias on the observed association. Second, the number of ANC visits were recalled by the mother who may have underestimated or overestimated the actual number of ANC visits. This may lead to a possible measurement misclassification bias, with subsequent under- or overestimation of the association between frequency of ANC visits and complementary feeding practices. Third, we were unable to assess all potential confounding factors (such as the type of local foods and cultural practices relating to IYCF, health worker’s knowledge of IYCF practices or family support systems and dynamics). This may have an impact on the association between the exposure and outcome variables. Lastly, the establishment of temporal association in this study is impossible given the use of cross-sectional data, where both the exposure and outcome variables were collected concurrently.

Despite these limitations, the study has strengths. Our study used the most recent and nationally representative data (NFHS-4) for India. The NFHS-4 data were obtained from a larger sample compared to previous national surveys, indicating that our findings are more representative of the Indian population in guiding up-to-date and evidence-based policy interventions. The data used are comparable across regions in India given that they were collected by trained personnel who used standardized questionnaires and methodology. The study findings are unlikely to be affected by selection bias as the survey yielded high responses rates, over 94%.

## Conclusion

The study found regional variations in prevalence and factors associated with complementary feeding indicators across India. Southern region followed by the North Eastern region had a higher prevalence of complementary feeding practices compared with other regions. Mothers with higher education, those from wealthier households and mothers who made frequent ANC visits were more likely to have appropriate complementary feeding practices compared with mothers with no schooling, those from poorer households and mothers who made no ANC visits across Indian regions. This study suggests that efforts to improve complementary feeding practices must be context-specific and should target mothers with vulnerabilities, including those from poorer socioeconomic backgrounds and no ANC visits. Findings from this study would assist policy decision-makers and public health managers in resource allocation to improve complementary feeding practices in India.

## Data Availability

The study was based on the 2015–16 India Demographic and Health Survey data, with some restriction imposed by the DHS program. Approval to use these data was sought from Measure DHS/ICF International, and permission was granted for this use. The data are available to apply for online at https://dhsprogram.com/data/available-datasets.cfm. Contact information for data access: The DHS Program Office, ICF, 530 Gaither Road, Suite 500, Rockville, MD 20850. Tel: + 1 301 407–6500; Fax: + 1 301 407–6501; email: info@dhsprogram.com

## Abbreviations

DHS: Demographic and health survey
ICF: Inner city fund
IIPS: International Institute for Population Sciences
IYCF: Infant and Young Child Feeding
MAD: Minimum acceptable diet
MAD: Minimum dietary diversity
MMF: Minimum meal frequency
NFHS: National Family and Health Survey
SDG: Sustainable development goal
TBA: Traditional birth attendant
UNICEF: United Nations Children’s Fund
WHO: World Health Organization
